# Progress in Studying Salt Secretion from the Salt Glands in Recretohalophytes: How Do Plants Secrete Salt?

**DOI:** 10.3389/fpls.2016.00977

**Published:** 2016-06-30

**Authors:** Fang Yuan, Bingying Leng, Baoshan Wang

**Affiliations:** Key Lab of Plant Stress Research, College of Life Science, Shandong Normal UniversityJi’nan, China

**Keywords:** asymmetric ion and water transport, recretohalophyte, salt gland, salt secretion mechanism, salt stress

## Abstract

To survive in a saline environment, halophytes have evolved many strategies to resist salt stress. The salt glands of recretohalophytes are exceptional features for directly secreting salt out of a plant. Knowledge of the pathway(s) of salt secretion in relation to the function of salt glands may help us to change the salt-tolerance of crops and to cultivate the extensive saline lands that are available. Recently, ultrastructural studies of salt glands and the mechanism of salt secretion, particularly the candidate genes involved in salt secretion, have been illustrated in detail. In this review, we summarize current researches on salt gland structure, salt secretion mechanism and candidate genes involved, and provide an overview of the salt secretion pathway and the asymmetric ion transport of the salt gland. A new model recretohalophyte is also proposed.

## Introduction

Soil salinization has long been known as an environmental problem (Jacobsen and Adams, 1958), and approximately 6% of the planet’s total land, or more than 800 million ha, is affected (Shabala, 2013). Forty-five million ha (20%) of presently irrigated lands are also saline lands (Munns and Tester, 2008; FAO, 2015; Shelden et al., 2016). In China alone, more than one million acres of agricultural land are salt affected due to irrigation water containing high soluble salts^1^. Moreover, secondary salinization caused by inappropriate irrigation is increasing in many countries, and it is difficult to reclaim land once degraded in this way despite the availability of substantial funding for land recovery. Few crops can survive in salt-affected regions, leading to substantially reduced production and often further degradation and desertification (Flowers and Colmer, 2008). Halophytes are considered promising species for the use and improvement of saline land (Song and Wang, 2014).

**FIGURE 1:**
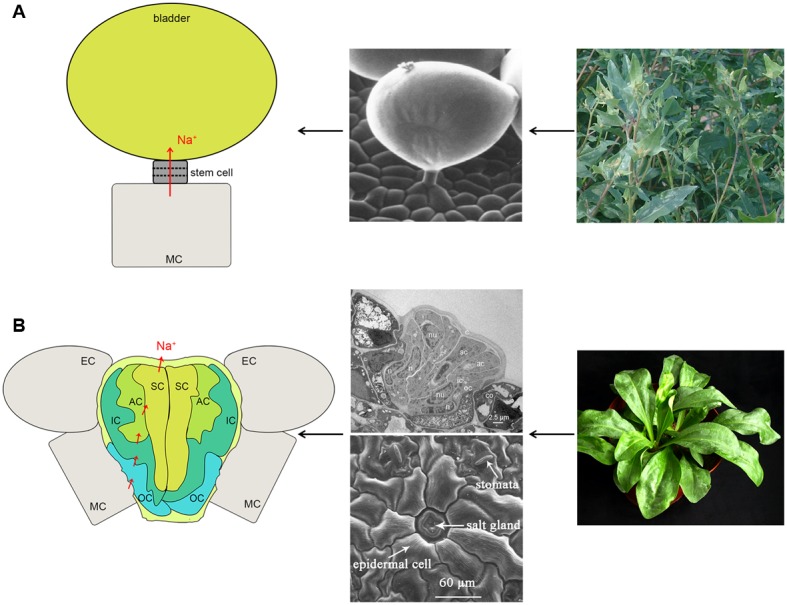
**The structure and Na^+^ secretion pathway of a salt bladder (A) and a salt gland (B). (A)** The large balloon represents the typical structure of the salt bladder. Na^+^ can be transported into the balloon and released after bladder rupture. The representative plant is *Atriplex centralasiatica*. **(B)** The typical multi-cellular salt gland and the Na^+^ pathway. The representative plant is *Limonium bicolor*. The photographs in **(B)** are reproduced from Feng et al. (2014) and Yuan et al. (2015) with some modifications. SC, secretory cell; AC, accessory cell; IC, inner cup cell; OC, outer cup cell; MC, mesophyll cell; EC, epidermal cell.

Halophytes, which constitute 0.4% of the total plants in the world, are plants that can survive and complete their life cycle in media containing more than 200 mM NaCl (Flowers and Colmer, 2008; Santos et al., 2015). Amongst these halophytes there is a small number that are able to secrete salt from their leaves, the so-called recretohalophytes (Flowers et al., 2015). There are approximately 370 recretohalophyte species all over the world according to statistics from Breckle (1995), Zhou et al. (2001), Flowers and Colmer (2008), and Flowers et al. (2010). Recretohalophytes are distributed widely around the globe, inhabiting seawater and inland saline lands (eHALOPH^2^). Salt-secreting structures, namely salt bladders (**Figure 1A**) and salt glands (**Figure 1B**), are the unique structures that directly secrete these ions out of the plant, and they are also notable for their presence in recretohalophytes and absence from other halophytes and all non-halophytes (Shabala et al., 2014; Yuan et al., 2015).

Salt bladders and salt glands differ in their structure. Salt bladders are composed of one bladder cell, without or with one or more stalk cells while salt glands consist of either two- or multi-cellular structures (the details are discussed in the section, The Reported Recretohalophytes and the Structural Characteristics of Salt Glands). Single epidermal cells can function as a salt bladder, as seen in *Mesembryanthemum crystallinum* and bladders are often modified trichomes. Salt bladders once differentiated, expand rapidly and after exposure of the plant to salt may break up releasing ions to the environment. Salt glands form stable structures that directly secrete salt out of the plant to the external environment.

The earliest studies on salt secretion were performed on the salt bladders of *Hormosira banksii* (Bergquist, 1959) and the salt glands of *Spartina townsendii* (Skelding and Winterbotham, 1939). Since the latter half of the 20th century, more investigations on the ultrastructure and salt secretion of recretohalophytes have been performed. In recent decades, remarkable progress has been made in explaining salt exclusion and secretion mechanisms and the development of salt bladders and salt glands, with most studies concentrating on two plants, *Chenopodium quinoa* and *Limonium bicolor*. *C. quinoa* is a typical recretohalophyte that possesses salt bladders, and its salt secretion mechanism and salt transport pathway were illustrated in detail in a recent review (Shabala et al., 2014). Comparison of metabolic changes in salt-treated relative to control samples without NaCl treatment showed that 352 different metabolites were identified in bladder cells of *M. crystallinum* under salt treatment (Barkla and Vera-Estrella, 2015). Recent studies of Oh et al. (2015) presented a transcriptomic analysis of bladder cells of *M. crystallinum* demonstrating cell-type-specific responses during adaptation to salt. The latest study of *Atriplex canescens* showed that the increasing of Na^+^ accumulation in salt bladders can enhance the salt tolerance (Pan et al., 2016). *L. bicolor* has multicellular salt glands and the mechanisms of development and salt secretion, in particular the candidate genes, have been studied (Feng et al., 2014; Yuan et al., 2015): more detail is provided below.

The topics of salt glands and salt secretion have been previously reviewed and details of publications prior to 2010s can be found in Flowers and Colmer (2008), Ding et al. (2010b), and Flowers et al. (2010, 2015). In the current review, in addition to the basics before 2010s, we mainly focus on salt secretion mechanisms in salt gland in recretohalophyte that were published in the last 5 years.

## The Reported Recretohalophytes and the Structural Characteristics of Salt Glands

**FIGURE 2:**
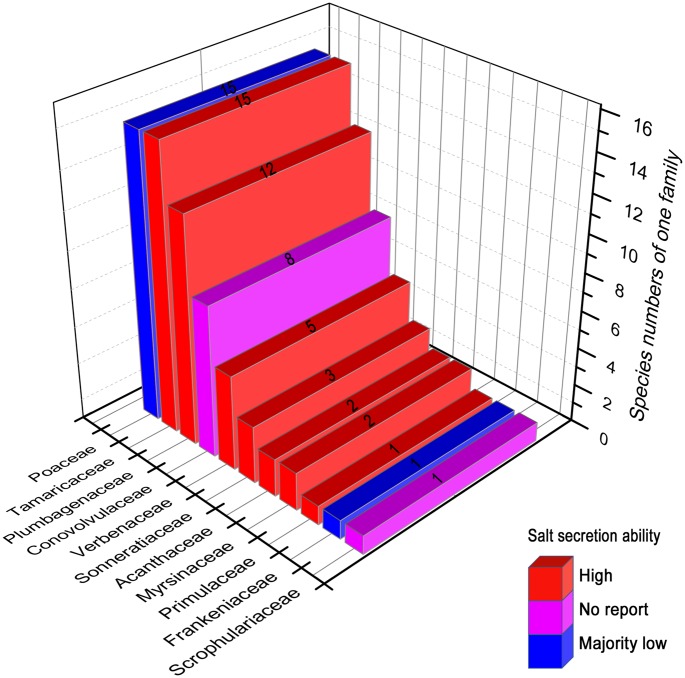
**The reported recretohalophytes possessing salt glands with different salt secretion ability.** The numbers on the bars presented the species numbers of one family. Red, the species of these families showed strong salt secretion. Purple, there has been no report about the salt secretion in these families until now. Blue, majority of this family showed week salt secretion except *Aeluropus*, *Sporobolus*, and *Spartina*. The figures were drawn with reference to Zhou et al. (2001) and Zhao et al. (2002).

To date, the following 11 families (65 species) have been discovered to have salt gland structures (**Figure 2**): Scrophulariaceae (one species; and the following number in parenthesis after each family represents the number of recretohalophyte species reported in that family), Frankeniaceae (1), Primulaceae (1), Myrsinaceae (2), Acanthaceae (2), Sonneratiaceae (3), Verbenaceae (5), Conovolvulaceae (8), Plumbagenaceae (12), Tamaricaceae (15), and Poaceae (15) according to the statistics of Zhou et al. (2001), Zhao et al. (2002), and Flowers et al. (2010). Most species were reported to have strong salt-secreting abilities as shown in **Figure 2**, e.g., *Frankenia grandifolia* of Frankeniaceae (Balsamo and Thomson, 1993), *Glaux maritima* of Primulaceae (Rozema and Riphagen, 1977), *Aegiceras corniculatum* of Myrsinaceae (Ball, 1988; Parida et al., 2004), *Acanthus ilicifolius* of Acanthaceae (Ye et al., 2005), *Sonneratia caseolaris* of Sonneratiaceae (Shan et al., 2008), *Avicennia marina* of Verbenaceae (Ball, 1988; Chen et al., 2010), *L. bicolor* of Plumbagenaceae (Ding et al., 2010a), and *Reaumuria trigyna* of Tamaricaceae (Dang et al., 2013). In the Poaceae, most genera showed low salt secretion ability except *Aeluropus* (Pollak and Waisel, 1970; Barhoumi et al., 2007), *Sporobolus* (Ramadan, 2001), and *Spartina* (Bradley and Morris, 1991). Attempts have been made to link the structure of salt gland (Zhao et al., 2002) to the salt secreting ability in two other families (Scrophulariaceae and Conovolvulaceae), and more findings involved in secretion ability will likely be discovered in both families in the near future.

The salt glands in different species possess various structural characteristics. The number of component cells has been used to separate multi-cellular salt gland and bi-cellular salt gland (**Figure 1**). In general, the salt glands in dicotyledonous recretohalophytes are multi-cellular and sunken into the epidermis. For instance, eight cells were identified in *Tamarix aphylla* with six secreting cells and two collecting cells in a symmetrical structure (Thomson and Platt-Aloia, 1985). Similarly, in *L. bicolor*, the salt glands consist of 16 cells, with four groups each of outer cup cells, inner cup cells, accessory cells and secretory cells (**Figure 1B**; Ding et al., 2010a; Feng et al., 2015; Yuan et al., 2015). Species of mangroves (of the Verbenaceae) grow in intertidal zone and possess salt glands with different numbers of secretory cells, e.g., 6–8 secretory cells in *A. officinalis* (Tan et al., 2010), 8–12 reported in *A. marina* (Shimony et al., 1973; Drennan et al., 1987), and eight found in *Avicennia germinans* (Balsamo and Thomson, 1993). In contrast to the multi-cellular glands, bi-cellular salt glands are found in the monocotyledonous recretohalophytes of the Poaceae, in species of *Aeluropus*, *Sporobolus*, *Spartina*, and *Zoysia* (see Ramadan and Flowers, 2004; Chen et al., 2009; Semenova et al., 2010; Céccoli et al., 2015). In all of the above examples, the innermost cells of the salt glands were positioned adjacent to the mesophyll cells, e.g., the collection cells in *Tamarix* and the outer cup cells in *Limonium*.

An interesting feature of salt glands is their autofluorescence under UV excitation (e.g., of *L. bicolor;* Yuan et al., 2013). By successfully isolating the salt gland complex *in vitro*, Tan et al. (2010) showed that in *A. officinalis* this phenomenon was produced by the cuticles around the salt gland and that these complexes are acidic in nature. Recently, Deng et al. (2015) used Sudan IV staining to show that the autofluorescent substance was localized in the cuticle of the salt glands, and simultaneously suggested that the ferulic acid in the cuticle was directly involved in the salt secretion of the *L. bicolor* salt gland. The cuticle was considered an essential structure for preventing leakage of ions and for protecting the mesophyll from salt damage. The detailed roles of autofluorescencing substances and the cuticle in salt secretion are the subjects of ongoing study.

## Comparison Between Different Methods for Measuring Salt Secretion

**FIGURE 3:**
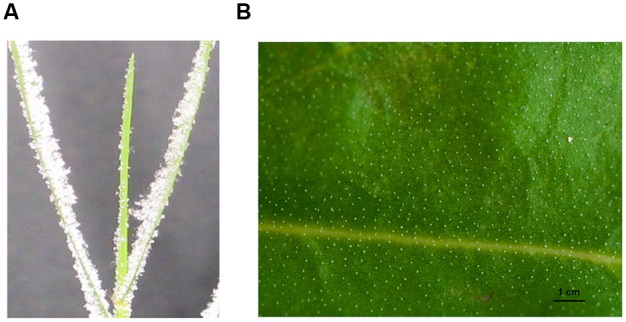
**The salt secretion of the salt gland with a distinct salt crystals on the leaves of *Distichlis spicata* (A) and *Limonium bicolor* (B).** The photograph in **(A)** was reproduced from Semenova et al. (2010). The leaf blades of *D. spicata* exhibited salt crystals on their leaf surfaces after the plants were incubated in 0.55 mM NaCl solution for 20–24 h under conditions that prevented the slightest air flow (Semenova et al., 2010). The photograph in **(B)** was reproduced from Feng et al. (2014). Leaves of *L. bicolor* that were treated with 200 mM NaCl displayed distinct salt crystals on the surface of leaves, and the salt secretion rate per single salt gland was 8 ng h^–1^, which is equivalent to a 5 mg Na^+^ secretion from a single mature leaf within 24 h after it was treated with 92 mg NaCl (200 mM) per day.

The salt secretion activity of a salt gland can be observed with the naked eye (e.g., **Figure 3**) or measured using leaf disks (Campbell et al., 1974; Thomson, 1975), a methodology that was recently improved by the use of oil (Yuan et al., 2013). In *L. bicolor*, in total 5 mg Na^+^ secretion on single mature leaf in 24 h, treated with 92 mg NaCl (200 mM) each day. By brushing the salt bladders from the surface of leaves in *A. canescens*, the Na^+^ concentration in bladders significantly raised with the increasing of external NaCl (Pan et al., 2016). However, these methods do not provide direct evidence of salt secretion by a single salt gland, so credible techniques are required in order to determine the salt secretion of a single salt gland. Such methods have been developed over the last 20 years (**Table 1**). X-ray microanalysis was first applied to *Porteresia coarctata* (Flowers et al., 1990) and *T. aphylla* (Storey and Thomson, 1994), and this method showed that the salt gland secreted Ca, Mg, and S as well as Na and Cl. Later, in the salt-secreting mangrove *A. marina*, X-ray fluorescence was used to determine the elemental composition with respect to Na, Cl, K, S, Ca, Br, and Zn (Sobrado and Greaves, 2000). SNP (sodium nitroprusside, a NO donor) significantly increased, particularly the Na^+^-to-K^+^ ratio in the salt glands of *A. marina* as measured by X-ray (Chen et al., 2010). Until recently, X-ray microanalysis was an accurate tool for measuring salt gland secretion and the position of the elements in the salt gland.

**Table 1:** Comparative summary for different methods to measure salt secretion in recretohalophyte.

Method	Principle of the method	Advantages	Disadvantages	Secreted ions and concentration	Possible driving forces	Applications in recretohalophytes
Leaf disks secretion covered with oil	The method was first developed by Faraday et al. (1986) and Dschida et al. (1992), and recently improved by the use of oil (Yuan et al., 2013). The secreted ion solution by salt glands on single leaf can be isolated in oil droplets. The accurate ion concentration can be obtained in the further steps.	Simple method without a need of special equipment, and usually costs 12–24 h. Resolution is at the level of whole plant or the single leaf.	Cannot provide direct evidence of salt secretion by a single salt gland.	Na^+^ 20–200 mM, K^+^ 5–20 mM, Mg^2+^ 4–11 mM, Ca^2+^ 0–10 mM, Cl^–^ 20–200 mM, et al.	HKT1, CNGC, NSCC, H^+^/Cl^–^ symporter	*Limonium bicolor* (Faraday et al., 1986; Yuan et al., 2013); *Avicennia germinans* (Dschida et al., 1992)
X-ray microanalysis	In X-ray analysis, the ratio of intensity of a target element of unknown concentration to that of an internal standard of known concentration is related to the concentration of the target element. The relative elemental sensitivity of the spectrometer was determined by the analysis of a multi-elemental aqueous standard solution containing V, Co, Zn, Se, and Sr (Sobrado and Greaves, 2000).	The method allows determinations in both materials from plants and animal species and is a useful tool because it provides multi-elemental analysis simultaneously.	Requires special equipment and strict procedure.	Na^+^ 20–200 mM, K^+^ 5–10 mM, Ca^2+^ 1–3 mM, Mg^2+^ 2–6 mM, SO_4_^2-^ 2–6 mM, et al.	HKT1, CNGC, NSCC	*Porteresia coarctata* (Flowers et al., 1990); *Tamarix aphylla* (Storey and Thomson, 1994); *Avicennia marina* (Sobrado and Greaves, 2000; Chen et al., 2010)
Method combined scanning electron microscopy (SEM) with X-ray	The method is based on SEM and X-ray analysis. SEM can give the clear component cells of salt glands and the secreted salts on the salt gland. In addition, X-ray can measure the ions by salt secretion in the specified locations. The structure–function relationships can be explored in-deep.	The method can give the intuitive secreted salt drops without any need for sectioning. Besides, this combined means may give the real-time secretion status of a salt gland. This is the most precise and accurate means to observe salt secretion and measure secreted ions.	Due to the complicated sample preparation procedures, this method required more accurate and efficient operations in order to measure salt secretion rapidly and precisely.	Na^+^ 20–200 mM, K^+^ 5–20 mM, et al.	HKT1, CNGC	*Chloris gayana* (Oi et al., 2013); *Cynodon dactylon* (Parthasarathy et al., 2015); *Limoniastrum guyonianum* (Zouhaier et al., 2015)
Non-invasive Microsensing System (NMS)-BIO-001A (Younger USA Sci.&Tech)	The ions can be measured by moving an electrode repeatedly between two positions in a predefined excursion (5–30 μm) at a programmable frequency in the range 0.01–10.00 Hz with a range of 0.3–0.5 Hz being typical for many types of electrodes.	The measurement of salt secretion of single salt gland is realized by this method. The salt secretion can be detected real-time. Most of inorganic ions can be measured, usually 5–20 min to measure one ion with simple operation.	NMS detects the net flux of an ion of a salt gland rather than ion efflux. Most of the organic acid anions cannot measure. Salt gland may be destroyed during peeling the epidermis.	Na^+^ 20–200 mM, K^+^ 5–20 mM, Cl^–^ 20–200 mM, et al.	HKT1, H^+^/Cl^–^ symporter	*Avicennia marina* (Chen et al., 2010); *Limonium bicolor* (Feng et al., 2014)
Nanoscale secondary ion mass spectrometry (SIMS)	This method is operated combined with high-pressure freezing (HPF) and freeze substitution (FS). The bombardment of ions results in the ejection of charged atomic and molecular species from the surface layers of the sample. These secondary ions are then separated on the basis of their mass-to-charge ratio using a high-performance mass spectrometer, and are correlated with their spatial origin to form a chemical image (Smart et al., 2010).	The ions can be measured with spatial resolutions of better than 100 nm. The accurate ion distribution and concentration are showed on a chemical image based on TEM analysis. This method resolved the problems of ion position, distribution and content *in situ*.	More applications need to be attempted in salt secretion of salt glands. The complicated and strict operating procedures may limit the wide applications.	Na^+^ 20–200 mM, K^+^ 10–300 mM in nucleus of salt gland.	HKT1, CNGC	*Limonium bicolor* (Feng et al., 2015)

*The distinction between the methods is partially arbitrary. The contents are partially referred to Volkov (2015). HKT1, high-affinity K^+^ transporter 1; CNGC, cyclic nucleotide-gated cation channel; NSCC, non-selective cationic channel.*

Subsequently, methods combining scanning electron microscopy (SEM) with X-ray were rapidly developed to measure salt secretion by single salt gland. *Chloris gayana* was found to secrete salt through its bicellular salt glands without rupturing the cuticle on the cap cell (Oi et al., 2013). Besides, salt glands can be identified in SEM images of intact leaves of *Cynodon dactylon* without any need for sectioning (Parthasarathy et al., 2015), and the salt glands observed by SEM in *L. bicolor* were apparently covered by exudates (Feng et al., 2014), which ensured the accuracy of observation *in situ*. Subcellular structures of the salt gland were also available by SEM and X-ray: in *Limoniastrum guyonianum*, where accumulating cells contained numerous, large and unshaped vacuoles (Zouhaier et al., 2015). SEM can also show the real-time secretion status of a salt gland and provide an in-depth understanding of structure–function relationships in multicellular salt glands (Zouhaier et al., 2015). Over a period of time, SEM has been recognized as the most precise and accurate means to observe salt secretion. However, the use of SEM and X-ray microanalysis in the estimation of salt secretion is beset by complicated sample preparation procedures. Consequently, researchers required more accurate and efficient methods in order to measure salt secretion rapidly and precisely.

Over the past 5 years, a new method called Non-invasive Microsensing System (NMS)-BIO-001A (Younger USA Sci.&Tech) has been widely applied to measure the secretion of salt glands. Using NMS, Chen et al. (2010) found that SNP treatments significantly increased net Na^+^ efflux from the salt glands of *A. marina*. Additionally, the Na^+^ secretion rate from *L. bicolor* was obtained by NMS, which was greatly enhanced by NaCl treatment (Feng et al., 2014). NMS can realize the aim of measuring salt secretion of single salt gland *in vivo* and continuous measurement. NMS can detect the net flux of an ion of a salt gland. However, researchers have to peel the epidermis of leaves in order to detect the salt secretion of salt gland. Salt gland may be destroyed during peeling.

Recently, a new technology was introduced to determine the ion position in recretohalophytes, and it is known as nanoscale secondary ion mass spectrometry (SIMS) combined with high-pressure freezing (HPF) and freeze substitution (FS), which can achieve higher spatial resolution than NMT (Smart et al., 2010). SIMS was first performed in the recretohalophyte *L. bicolor*, and Feng et al. (2015) showed that K^+^, which accumulated in both the cytoplasm and nucleus of salt gland cells under salinity, may play an important role in salt secretion, although the exact mechanism is unknown. The using of SIMS resolved the problems of ion position, distribution and content *in situ*, but complicated and strict operating procedures limited its application.

In conclusion, methods are now available for the elemental analysis of secretions from salt glands. Now, the question arises as to how the ions are transported into the salt gland and the role played by the unique structure of salt glands in the secretory process.

## How is Salt Transported into the Salt Gland?

Ion transport is the essential factor determining salinity tolerance in plants (Volkov, 2015). Salt gland can secrete various ions, and the secreted elements mainly depend on the environment (Kobayashi et al., 2007). Amounts of inorganic elements were reported to be excluded by salt glands in the late 5 years including various kinds cationic elements (Na, K, Ca, N, Mg, Fe, Mn, Si, and Zn) and anionic elements (Cl, O, Br, S, P, and C; Sobrado and Greaves, 2000; Semenova et al., 2010; Oi et al., 2013; Feng et al., 2014, 2015; Céccoli et al., 2015; Parthasarathy et al., 2015; Zouhaier et al., 2015), but secretion of Na^+^ and Cl^–^ is higher than that of other ions (Ding et al., 2010b; Ma et al., 2011).

No direct evidence has been obtained to confirm the pathway of salt transport into the salt gland, but a number of possible paths have been suggested. The innermost cells of the salt glands were positioned adjacent to the mesophyll cells, the unique structures of salt glands determined the salt transported into the salt gland. A question then arises as to what caused salt to transfer from mesophyll cells into the salt gland directionally and without influence from the mesophyll cells. Which structure isolates the salt gland from the mesophyll? The cuticles surrounding the salt gland are likely to play this role.

Previous studies on the salt gland ultrastructure in *Spartina foliosa* (Levering and Thomson, 1971) and *T. aphylla* (Thomson et al., 1969) demonstrated that cuticles were present around the salt glands, and they formed a thick barrier from the mesophyll and the external environment. New findings of *Distichlis spicata* showed that these ions were transported into the salt gland through the bottom penetration area that was not covered by the cuticles of the salt gland, and the cuticles can prevent the ions from backflowing into the mesophyll (Semenova et al., 2010). Ions accumulated in the salt gland via the bottom penetration area and plasmodesmata generated fluid pressure due to the presence of the cuticle, and then secreted through salt gland pores. As stated in the former section, ferulic acid in the cuticle was proved to be directly involved in the salt secretion of salt glands (Deng et al., 2015). Therefore, cuticles play a significant role in salt secretion.

The plasmodesmata were considered as another typical feature in the ultrastructure of salt gland (Thomson and Platt-Aloia, 1985). Apparently, the plasmodesmata were present as a membrane-lined intercellular bridge between the mesophyll cells and the salt gland, which allowed these plant cells to communicate with virtually all the adjoining cells (Sager and Lee, 2014). The plasmodesmata were assumed to play an essential role in ion transport into the salt gland (Faraday et al., 1986). The callose and lipid are the most important composition of plasmodesmata, which are well-illustrated to be necessary for ion transport (Grison et al., 2015). Plasmodesmata showed dynamic structures that can actively and selectively transport very large molecules between cells (Overall and Blackman, 1996). However, the detailed role and mechanism of the plasmodesmata in salt gland ion transport required further verifications.

Of equal importance, large central vacuoles were not present in the salt gland cells; instead, many vesicles were detected (Thomson and Platt-Aloia, 1985; Tan et al., 2010, 2013). In particular, the neighboring mesophyll cells had much larger vacuoles than normal, and the secretory cells of the salt glands adjacent to the mesophyll cells possessed many small vesicles, such as *Limonium* (Ziegler and Lüttge, 1967; Yuan et al., 2015; Zouhaier et al., 2015). These small vesicles may play an important role in transporting salt into the salt gland.

**FIGURE 4:**
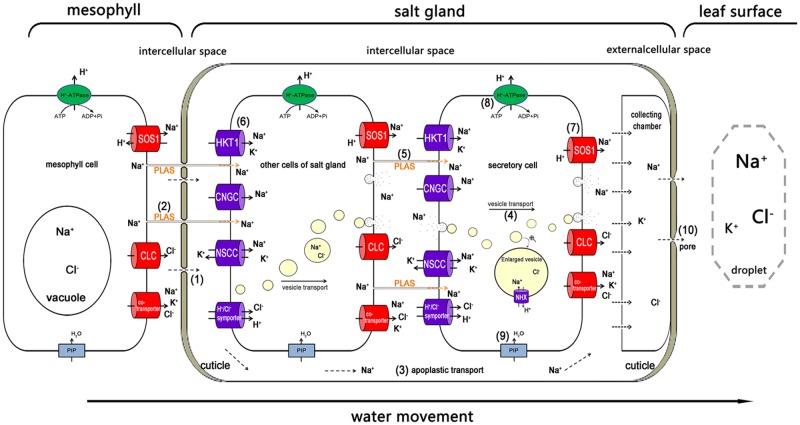
**The possible pathway of Na^+^ transport from the mesophyll to the salt gland, and the way in which Na^+^ is secreted out.** Na^+^ is transported into the salt gland through the bottom penetration area, which is not covered with cuticles (1), and the plasmodesmata (2). In the salt gland, the ions can be directly transported into the intercellular space (3). The ions are wrapped in vesicles for transport from the cytosol to the plasma membrane and secreted out of the salt gland cells (4). Plasmodesmata play an important role in ion delivery among the salt gland component cells (5). Various ion transporters are widely involved in moving salt in (6) and out (7). H^+^-ATPase participated in salt secretion by establishing electric potential difference and proton motive force across plasma membrane (8). Water channels (e.g., PIP) also take part in all the processes as a medium for ion transport (9). The ions are eventually exuded from the secreting pores at the top of the salt gland due to high hydrostatic pressure (10). Ion transporters responsible for influx (dark blue) and efflux (red) are asymmetrically distributed in the plasma membrane of salt gland cells. PLAS, plasmodesmata; HKT1, high-affinity K^+^ transporter 1; CNGC, cyclic nucleotide-gated cation channel; NSCC, non-selective cationic channel; PIP, plasma membrane intrinsic protein; NHX, Na^+^/H^+^ antiporter; SOS1, Na^+^/H^+^ antiporter; CLC, chloride channel.

In addition to the unique structural characteristics, the membrane-bound translocating proteins are likely to be involved in salt secretion. Another significant pathway passed through the ion carriers or channels inside the plasma membrane of the salt glands, which were similar to those in normal cells with possible exception that these transporters mainly distributed asymmetrically in the other side of plasma membranes of salt gland cells adjacent to the collecting chamber (**Figure 4**), e.g., the potassium transporter (HKT1 and KUP), the inward rectifier potassium channel (KEA), with the CNGC and NSCCs eventually increasing the Na^+^ accumulation in the cytoplasm of the cells (Flowers et al., 1977; Flowers and Colmer, 2008). A low-affinity K^+^ transporter called AlHKT2;1 from the recretohalophyte *Aeluropus lagopoides* played an important role in K^+^ uptake during salt stress and in maintaining a high K^+^/Na^+^ ratio in the cytosol (Sanadhya et al., 2015b).

Above all, salt was mainly transported into the salt gland through the plasmodesmata, vesicle transport and ion transporters, which combined with both apoplastic and symplastic transport. Even so, there is still a question as to what mechanism drives the salt out of the salt gland.

## A Possible Pathway for Secreting Salt from the Salt Gland

As stated above, salt secretion was believed to be an active physiological process of the plant. Various physiological data indicated that salt secretion activity requires large amounts of energy. Salt secretion was typically higher under light treatment than it was in the dark (Dschida et al., 1992). Under salinity, the expression of H^+^-ATPase in particular increased with the same trend as that of the salt secretion (Chen et al., 2010). However, as reported in *T. aphylla* and *L. bicolor*, no chloroplast was present in the salt gland (Thomson and Liu, 1967; Shimony and Fahn, 1968; Yuan et al., 2015). Therefore, the plasmodesmata between the mesophyll cells and the salt glands in *L. bicolor* may provide the salt gland with photoassimilates and NADPH from the mesophyll cells, which were the original sources of energy for salt excretion. Moreover, during the differentiation of salt glands, mitochondria are the first subcellular organelle differentiated to provide the energy for differentiation of salt gland with small folding mitochondrial crista. The mitochondria in salt glands showed larger scale than that in mesophyll cells (Yuan et al., 2015).

Apparently, all ions are transported out of the salt gland by water; therefore, water efflux is indispensable for salt exclusion. Two aquaporin genes (PIP and TIP) were preferentially expressed in the salt gland cells of *A. officinalis*, and aquaporins were thought to contribute to the re-absorption of water during salt removal (Tan et al., 2013).

Three hypotheses have been proposed to explain the salt secretion of recretohalophytes on the basis of the ultrastructure, and Ding et al. (2010b) have discussed this progress in some detail which rely on (1) the role of the osmotic potential in salt secretion in *Limonium latifolium* (first suggested by Arisz et al., 1955), (2) a transfer system in *S. foliosa* that is similar to liquid flow in animals (raised by Levering and Thomson, 1971) and (3) salt solution excretion by vesicles in the plasma membrane (exocytosis; promulgated by Ziegler and Lüttge, 1967; Shimony and Fahn, 1968).

In the current paper, we concentrate on the newest findings on salt secretion that have been uncovered over the last 5 years; interested readers can obtain details of previous results in Ding et al. (2010b). The recent findings can be separated into three areas consistent with the previous studies. (1) As far as the first suggestion is concerned, recent finding from a study of *D. spicata* showed that the parietal layer of the cytoplasm is invaginated into the extracellular space (apoplast), which is separated only by a thin single membrane. A series of vacuolar-apoplastic continua were identified, and they look like a valve (Semenova et al., 2010). Ions are separated in the salt gland by cuticles and then secreted through secreting pores by fluid pressure. (2) Ouabain, a specific Na^+^-ATPase inhibitor, can inhibit Na^+^ efflux and enhance K^+^ influx by combination with the outside K^+^ binding sites (Kim et al., 2013; Mette et al., 2015). Comparisons with animal transport systems have shown that in *C. gayana* (Kobayashi et al., 2007) and the *Tamarix* species (Ma et al., 2011), salt secretion from their salt glands was significantly decreased in the presence of ouabain, which acts as a salt secretion inhibitor and indicates the existence of liquid flow in salt gland (possible Na^+^-ATPase). (3) In terms of exocytosis, electron-dense substances primarily accumulated in the vesicles of the salt gland in *L. bicolor*, which showed that under salt treatment, numerous vesicles fused with the plasma membrane (Feng et al., 2014; Yuan et al., 2015). While all three hypotheses have support from studies of different recretohalophytes, in the last few years, more and more evidence has supported the role of exocytosis (Echeverría, 2000). TEM images showed an identifiable vesicle-transporting pathway in the salt gland of an *L. bicolor* leaf that was prepared by HPF, followed by FS and staining (Feng et al., 2015).

Additionally, ion transporters were proposed as another important pathway for salt glands to secrete Na^+^. H^+^-ATPase may participate in salt secretion through establishing electrochemical potential difference and proton gradient across plasma membrane of salt gland. Leaf H^+^-ATPase activity and the photosynthetic capacity of *Cakile maritima* can enhance salt resistance under increasing salinity (Debez et al., 2006). H^+^-ATPase and the Na^+^/H^+^ antiporter are anticipated to have roles in the salt secretion and Na^+^ sequestration of *A. marina* (Chen et al., 2010; Tan et al., 2010). Currently, high-throughput sequencing technology has broad and increasing applications for many recretohalophytes, and it has validated the view that various types of transporters may participate in salt secretion. Transcript profiling in *A. lagopoides* revealed that HAK, SOS1, and V-ATPase genes play a key role in regulating ion homeostasis (Sanadhya et al., 2015a). NHX and other members of the family play important roles in K^+^ homeostasis, vesicle trafficking and cell expansion (Rodríguez-Rosales et al., 2009). The expression of SOS1 and NHX1 was proportional to the salt secretion in *A. marina* (Chen et al., 2010).

Recently, the application of transriptomics in recretohalo phytes enhances the discovery of candidate genes involved in salt secretion. The transcription profiles of the recretohalophyte *R. trigyna* indicated that the genes related to ion transport were relevant to the salt secretion function of this species (Dang et al., 2013, 2014). Similarly, the candidate genes encoding ion transporters were also suggested in *Sporobolus virginicus* by RNA-seq (Yamamoto et al., 2015). An NaHCO_3_-treated *L. bicolor* cDNA library was established (Wang et al., 2008), and a recent RNA-Seq library containing the different developmental stages of *L. bicolor* provided an accurate resource for identifying the genes encoding ion carriers and transporters (Yuan et al., 2015).

Nowadays, proteomics was gradually applied to investigate salinity responsive proteins in three recretohalophytes. A cell-type-specific proteomics approach was taken in individual epidermal bladder cells of *M. crystallinum* by shotgun peptide sequencing, with a high representation of proteins involved in H^+^-transport, carbohydrate metabolism, and photosynthesis (Barkla et al., 2012). Besides, Tan et al. (2015a) developed a reliable procedure for obtaining proteins from salt gland-rich tissues of *A. officinalis* and profiled the proteome through 1D-LC-MS/MS. Meanwhile, Tan et al. (2015b) used shotgun proteomics to identify proteins of *A. officinalis* that are present in the salt gland-enriched tissue by comparing the protein profiles of salt gland-enriched (isolated epidermal peels) and salt gland-deprived (mesophyll) tissues, which elucidated the molecular mechanism underlying secretion in plant salt glands. Using the suspension cell cultures of *Halogeton glomeratus*, iTRAQ-based proteomic approach was conducted to reveal several proteins involved in energy, carbohydrate metabolism, stress defense, protein metabolism, signal transduction, cell growth, and cytoskeleton metabolism (Wang et al., 2016). Transcriptomics combined with proteomics will play an essential role in explaining salt secretion of salt gland of recretohalophytes.

However, all the known transcriptomes and proteomes described above are constructed on the basis of extractions from plant leaves, dominated by mesophyll cells so that genes differentially expressed on salt treatment were very likely derived from mesophyll cells; thus, we cannot determine that these genes are specific to the salt gland unless salt glands are first isolated for sequencing. As mentioned previously, isolating a single salt gland is feasible by enzymolysis in *A. officinalis* (Tan et al., 2010). Additionally, laser capture microdissection (LCM) technology has been used for separating target cells or tissues in many species (Li et al., 2010). Both methods can be introduced to isolate sufficient amounts of salt glands of sufficient purity for sequencing in the future.

Another approach to understanding salt secretion by recretohalophytes would be to analyze mutants whose salt secretion is in some way modified. Though many mutants in salt secretion have been obtained using gamma ray irradiation (Yuan et al., 2013), no recretohalophyte where specific genes have been silenced has been developed. Until now, the experiments suggesting candidate genes have only been reported in heterologous expression, such as *Lbchi32* of *L. bicolor* verified in pathogenic fungi (Liu et al., 2010). So the mutants in specific and candidate genes should be investigated in the future in order to accurately study molecular mechanism of salt secretion.

## Conclusion and Perspectives

It is widely accepted that salt secretion is closely aligned with the structure of salt glands (Ding et al., 2010b). Although salt glands in different species possess varying characteristics, their common characteristics can be summarized as follows: a thickened cuticle surrounding the salt gland, frequent plasmodesmata, a large number of developed mitochondria, no chloroplasts, and amounts of small vesicles in the cytoplasm (Thomson and Platt-Aloia, 1985; Ding et al., 2010b; Tan et al., 2013; Yuan et al., 2015). This special structure determines the functionality of bladders and glands; an ion can be rapidly transported from a mesophyll cells into a salt gland and secreted out of the salt gland with a force that is generated by mitochondrial activity and is then transported in vesicles, eventually being excluded through pores (Feng et al., 2014).

Using the muti-cellular salt gland such as *L. bicolor* as the model, **Figure 4** shows the possible pathway for an ion (e.g., Na^+^) transport from the mesophyll cell into the salt gland and the pathway of ion secretion. Salts from roots are transported upward in the transpiration stream. The salt transport process that moves ions in and out of the salt gland is thought to consist of a combination of both apoplastic and symplastic transport (Flowers and Yeo, 1986; Yeo and Flowers, 1986). Most of the Na^+^ is transported into the salt gland through the basal penetration area that is not covered by cuticles (1), and prevented by the pull of transpiration stream and a valve structure only open inwardly (Semenova et al., 2010), so Na^+^ cannot reflux through the basal penetration area. In other regions of the salt gland, the cuticles can also prevent ions from penetration into the mesophyll. Another possible pathway of the Na^+^ flowing into the salt gland is the plasmodesmata (2). Once in the salt gland, there are two possible pathways for the movement of Na^+^. The ions can be directly transported into the intercellular space that is separated from the mesophyll cells by the cuticles (3), where they can be temporarily collected in the collecting chamber. The ions are wrapped in vesicles for transport from the cytosol to the plasma membrane of salt gland cells and for secretion out of the salt gland; the membrane of vesicles can be recycled into plasma membrane. NHX may play an important role in transporting Na^+^ into the vesicles (4). The plasmodesmata provide an important pathway for ion movement between the salt gland component cells (5). Various ion transporters and channels are widely involved in transporting Na^+^ and Cl^–^ in (6) (e.g., HKT1, CNGC, NSCC and H^+^/Cl^–^ symporter) and out (7) (e.g., SOS1, CLC and various types of co-transporters) of cells. Ions that are transported by symplastic transport are also temporarily stored in the collecting chamber. During the transport of ions in both import and export pathways, the chemical compounds such as photoassimilate is transported by the plasmodesmata, and ATP is directly generated from highly developed mitochondria (8). Water channels also participate as a medium for ion transport (9). The ions are eventually excluded from the secreting pores that are located in the cuticles in the top of the salt gland due to high hydrostatic pressure (10). The transport of ions from mesophyll cells into and out of salt glands is an asymmetric process, which is a common phenomenon in many biological systems, such as IAA polar transport (Estelle, 1998; Reinhardt et al., 2003), gravity-induced polar transport of calcium across root tips of maize (Lee et al., 1983), iron uptake of the root (Dubeaux et al., 2015), and etc. The asymmetric distribution of salt secretion components primarily involves the asymmetric ion uptake and efflux; asymmetry in the vesicle transport direction; and asymmetry in ion transporter distribution. Most ions are transported in the direction of the vesicles, and the ion transporters that are responsible for secreting ions out of the salt gland are primarily distributed along the outside of secretory cells.

The study of salt secretion in recretohalophytes has developed considerably in recent years. More evidence has been proposed to explain the salt secretion mechanism; however, uncertainties still exist. On one hand, the salt secretion activity is mediated by multi-cells and multi-genes, and thus, the underlying control of the salt gland development remains unclear. Although, Yuan et al. (2015) have illustrated five differentiation stages in the *L. bicolor* leaf and proposed that a series of candidate genes can participate in salt gland development and salt secretion (Yuan et al., 2016), the direct validations of the key genes are needed on the basis of these data. On the other hand, RNA-seq has been conducted in five recretohalophytes, but to date, no genome sequencing has been reported in any recretohalophyte. Unclear genetic background made molecular studies difficult, and a new proposed model recretohalophyte is urgently needed. Fortunately, as a typical recretohalophyte possessing salt glands, *L. bicolor* is a potential model plant for investigating the salt secretion mechanism by salt glands in the future. Partly deficient salt-gland mutants have been obtained by physical and chemical mutagenesis in *L. bicolor* (Yuan et al., 2013). Moreover, the mutant traits can be kept for long-term investigation because the seeds from these plants can be collected through self-pollination (Yuan et al., 2014b). Based on the established transformation system and leaf disk secretion model (Yuan et al., 2014a), the key genes involved in salt secretion can be transformed for validation. Efficient genome editing in plants using a CRISPR/Cas9 system in non-modal plants will also be used to study molecular mechanism of salt secretion of plant salt gland (Feng et al., 2013). For a decade, various ion transporters have been shown to participate in salt resistance as verified in *Arabidopsis* and *Eutrema/Thellungiella* spp. (Lee et al., 2016). However, the in-depth mechanism of halophyte salt tolerance is largely unknown. More and more scientists have begun to use halophytes as materials to study the salt tolerance mechanism. We believe that in the near future, the salt tolerance mechanism will be completely uncovered, and the dream of generating salt-tolerant crops will finally come true.

## Author Contributions

FY and BL wrote the manuscript. BW modified the article. All authors read and approved the final manuscript.

## Conflict of Interest Statement

The authors declare that the research was conducted in the absence of any commercial or financial relationships that could be construed as a potential conflict of interest.

## Footnotes

1 http://www.fao.org/home/en/

2 http://www.sussex.ac.uk/affiliates/halophytes/
